# Identification of Null Alleles and Deletions from SNP Genotypes for an Intercross Between Domestic and Wild Chickens

**DOI:** 10.1534/g3.113.006643

**Published:** 2013-08-01

**Authors:** Lucy Crooks, Örjan Carlborg, Stefan Marklund, Anna M. Johansson

**Affiliations:** * Department of Cell and Molecular Biology, Uppsala University, Uppsala, SE-75124, Sweden; † Department of Clinical Sciences, Swedish University of Agricultural Sciences, Uppsala, SE-756 51, Sweden; ‡ Department of Animal Breeding and Genetics, Swedish University of Agricultural Sciences, Uppsala, SE-75007, Sweden

**Keywords:** null allele, deletion, chicken

## Abstract

We analyzed genotypes from ~10K single-nucleotide polymorphisms (SNPs) in two families of an F_2_ intercross between Red Junglefowl and White Leghorn chickens. Possible null alleles were found by patterns of incompatible and missing genotypes. We estimated that 2.6% of SNPs had null alleles compared with 2.3% with genotyping errors and that 40% of SNPs in which a parent and offspring were genotyped as different homozygotes had null alleles. Putative deletions were identified by null alleles at adjacent markers. We found two candidate deletions that were supported by fluorescence intensity data from a 60K SNP chip. One of the candidate deletions was from the Red Junglefowl, and one was present in both the Red Junglefowl and White Leghorn. Both candidate deletions spanned protein-coding regions and were close to a previously detected quantitative trait locus affecting body weight in this population. This study demonstrates that the ~50K SNP genotyping arrays now available for several agricultural species can be used to identify null alleles and deletions in data from large families. We suggest that our approach could be a useful complement to linkage analysis in experimental crosses.

Null alleles at genetic markers are alleles that do not amplify during genotyping. They can be due to a deletion encompassing the marker, polymorphisms in the sequence where the primer should anneal or a single-nucleotide polymorphism (SNP) site being triallelic. Individuals that are heterozygous for a null allele often are genotyped as homozygous for their other allele. Consequently, null alleles can create incompatibilities between the genotypes of parents and offspring. Genotype incompatibilities can also occur through genotyping error. Because incorrect genotypes will affect the results of genetic analyses, markers showing incompatible genotypes often are excluded or some genotypes changed to missing values. However, identifying null alleles can be valuable for discovering functional variation. In particular, null alleles are an indication of a possible deletion. In humans, incompatibilities and missing values in genotype data from high-density SNP arrays (~1 million SNPs) have been used to map hundreds of deletions in the range of 1−1000 kb (Conrad *et al.* 2006; McCarroll *et al.* 2006; Kohler and Cutler 2007).

Copy number variation, which includes deletions, has been recognized as an important component of human genetic diversity in the last few years. McCarroll *et al.* (2008b) detected 3048 regions with copy number variants (CNVs) from 270 people with a hybrid SNP-CNV array (1.8 million sites). Most of the CNVs that were in more than one individual were 1–500 kb long and they totaled 1.1% of the genome. Conrad *et al.* (2010) found and validated 8,599 CNVs from 41 people by array comparative genome hybridization (42 million probes). The majority of these CNVs were also 1−500 kb long and they covered 3.7% of the genome. CNVs have been associated with several complex human diseases (Girirajan *et al.* 2011). For example, a common 20-kb deletion upstream of the *IRGM* gene is a possible causal factor in Crohn’s disease (McCarroll *et al.* 2008a). In the future, genome-wide association studies may be extended to CNVs (Craddock *et al.* 2010), and a challenge is to integrate CNVs with SNPs and smaller insertions and deletions (indels) into combined genetic analyses (Korn *et al.* 2008).

Less is known about the prevalence of CNVs in most other species. In chickens, Wang *et al.* (2010) measured two to four individuals from each of three domestic breeds by array comparative genome hybridization (~400K probes). They found 96 CNVs with lengths from 10 kb to 2 Mb, which totaled 1.3% of the genome. Two studies have applied next-generation sequencing to CNV detection in chickens. Rubin *et al.* (2010) sequenced pooled samples for eight domestic chicken lines and Red Junglefowl at low depth and identified deletions by regions with no read coverage. They reported 1284 deletions, nearly all of which were shorter than 6 kb. One of the few longer deletions coincided with a QTL found in a cross between two of the lines and was associated with increased growth in the intercross offspring. Kerstens *et al.* (2011) performed paired-end sequencing on reduced representation libraries from pooled samples for four commercial chicken lines. They looked for paired reads mapping a significantly different distance apart than the expected insert size and discovered 188 CNVs. Of the deletions, most were smaller than 3 kb, and 40% were line specific. These studies indicate that deletions can contribute to genetic and phenotypic differences between chicken populations. However, the size range of deletions found by resequencing compared with the findings described previously for humans suggests that these sequencing approaches may be limited for detecting large deletions. Sequencing is also considerably more expensive than genotyping with microarrays. Although pooling samples reduces the cost, it provides no information on the CNVs in each individual.

Several traits in chicken have recently been shown to be caused by structural variations in the genome, demonstrating that it is necessary to look beyond point mutations as causative genetic factors. An 8-kb deletion upstream of *SOX10* is the causative mutation for the Dark brown plumage color (Gunnarsson *et al.* 2011). The frizzle feather trait is caused by a smaller deletion of 69 bp in a conserved region of *KRT75* (Ng *et al.* 2012). A complex rearrangement involving the *EDN3* locus produces the black pigmentation in Silkie chickens and three other breeds (Dorshorst *et al.* 2011). Two mutations affecting the shape of the comb have also been shown to be structural rearrangements. The Rose-comb is a result of an inversion on chromosome 7 (Imsland *et al.* 2012) and the Pea-comb is caused by a large number of copies of a duplicated sequence in intron 1 of *SOX5* (Wright *et al.* 2009).

Recently ~50K SNP arrays have become available for several agricultural species. Here, we explore the incidence of null alleles in genotype data of this density and their potential for finding deletions. An advantage of many animals and plants compared with humans for family-based null allele studies is the large number of offspring. There is stronger evidence for a null allele if genotype incompatibilities and missing values are seen for multiple individuals. The deletion studies in humans used only one offspring. We analyzed genotypes for just more than 10K SNPs in two chicken families from an experimental intercross. We distinguished five classes of possible null alleles and statistically assessed how well each class identified null alleles rather than genotyping error. Putative deletions were inferred by null alleles at adjacent markers. These were examined further by genotyping a subset of individuals with a 60K SNP chip. We discuss how applying this null allele method to intercross data could complement linkage analysis for identifying genetic factors underlying complex traits.

## Material and Methods

### Studied population

Individuals were from two three-generation families of a well-studied F_2_ intercross between White Leghorn and Red Junglefowl chickens (Kerje *et al.* 2003). The White Leghorn is a domestic layer breed with a much greater growth rate and body size than the Red Junglefowl, which is the major wild ancestor. The founders for the population were a single Red Junglefowl male and three White Leghorn females. There were four F_1_ individuals in the two studied families; they all had the same father and two also had the same mother. From these four F_1_ parents, two families were generated comprising 23 and 25 F_2_ individuals, respectively. Genotypes from 10,150 SNPs (Groenen *et al.* 2009) on autosomes 1−28 were examined (File S1). Physical positions for the 10K SNPs were taken from the Single-Nucleotide Polymorphism Database build 131 and for those not found in Single-Nucleotide Polymorphism Database, from the chicken genome assembly, build 2.1.

### Null allele detection

We wrote software to report SNPs with genotype incompatibilities or many missing values in the three-generation families. When a parent and offspring are genotyped as homozygous for different alleles and the other parent has the allele in the offspring, the offspring could have inherited a null allele from the first parent. When several offspring have missing values and both parents are genotyped as homozygous or missing, the missing genotypes could be the result of inheriting two null alleles. If there are different homozygotes in the offspring but no heterozygotes, one parent has a missing genotype and the other parent is heterozygous, the first parent could have two null alleles with all the offspring inheriting one null allele. The genotypes for all three generations had to be consistent with a null allele. For an F_1_ parent to have a null allele, one of its parents (grandparents) had to be homozygous or missing. The software is available from the authors on request.

### Classes of null alleles

**Figure 1:**
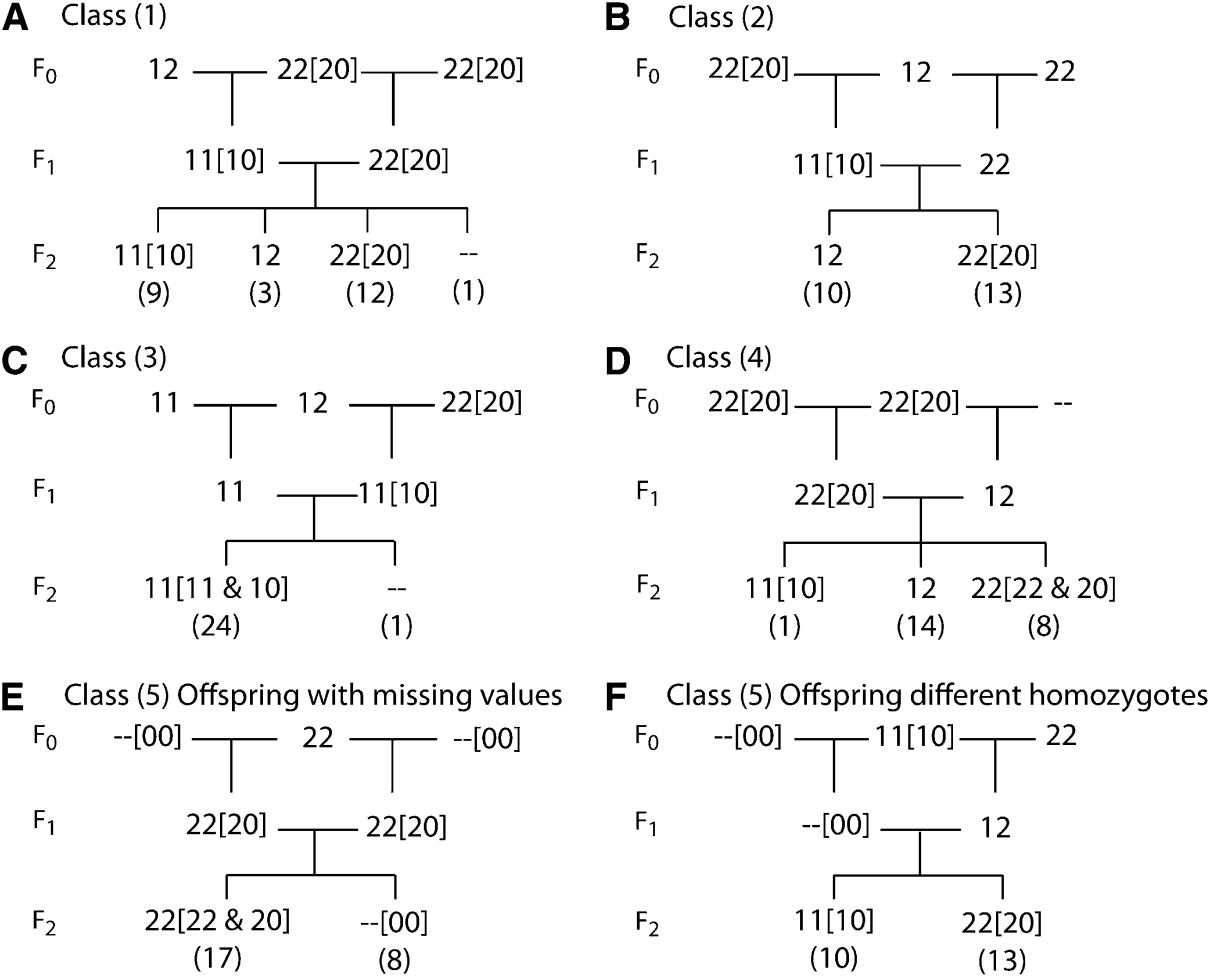
Examples of the five null allele classes. Examples are taken from the chicken data analyzed in this article (File S1) with the different alleles labeled 1 and 2. Missing genotypes are written as “--.” Null allele genotypes that could explain the observed genotypes are shown in square brackets with a null allele represented by 0, *i.e.*, the genotype 00 is homozygous for a null allele and the genotype 20 has one copy of allele 2 and one null allele. The number of F_2_ offspring with each genotype is given in round brackets. (A) A class (1) null allele. Here, the F_2_ offspring genotyped 22 are a different homozygote from the F_1_ parent genotyped as 11. They could have inherited a null allele from the first parent (transmitted from the first of its parents) and a 2 allele from the second parent. There are 22 instances in which a parent and offspring are genotyped as different homozygotes and three genotyping errors would be needed to explain these inconsistencies (each parent actually having genotype 12 and one of the parents of the second parent having genotype 11 or 12). (B) A class (2) null allele. There are 14 instances in which a parent and offspring are genotyped as different homozygotes. However, a single genotyping error could explain the inconsistencies (first F_1_ parent actually having genotype 12). (C) A class (3) null allele. The second F_1_ parent is a different homozygote from one of its parents. Because both F_1_ parents have the same homozygous genotype, no incompatibilities are expected in the F_2_ offspring. (D) A class (4) null allele. One F_2_ offspring is genotyped as a different homozygote from its parent. If there were a null allele, more offspring would be expected to show the same incompatibility. (E) A class (5) null allele. Multiple F_2_ offspring with missing values indicate the inheritance of a null allele from each parent. (F) Another class (5) null allele. F_2_ offspring are genotyped as different homozygotes and none are genotyped as heterozygotes. The first F_1_ parent could have two null alleles.

**Figure 2:**
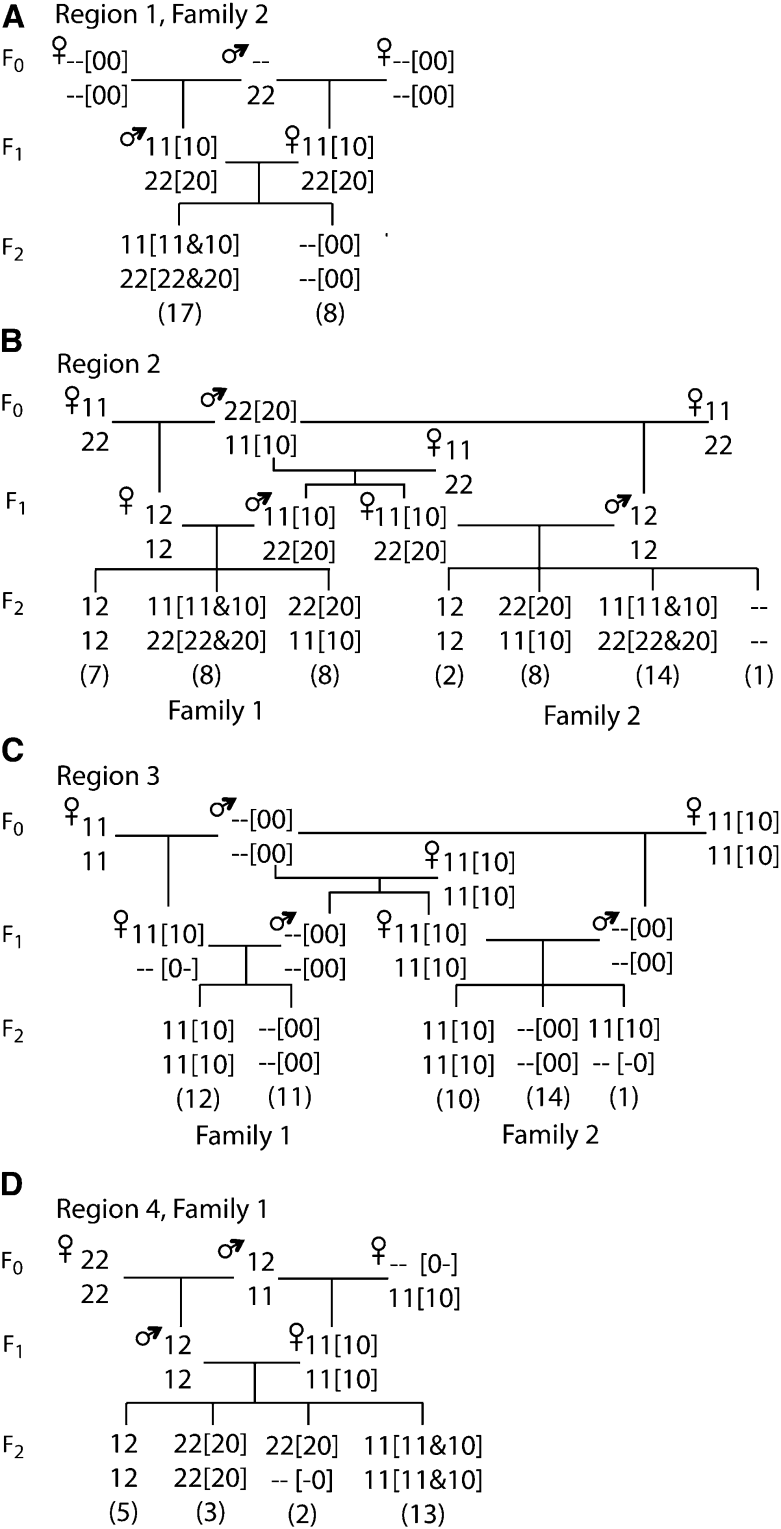
Genotypes at adjacent SNPs indicating putative deletions. Genotypes for the two SNPs are shown one above the other, with the first SNP on top. The number of F_2_ individuals with each genotype is shown in round brackets. Missing genotypes are written as “--.” Null allele genotypes that could explain the observed genotypes are shown in square brackets with a null allele represented by 0. Within the brackets “–” denotes an unknown allele. In region 1, the null allele was assumed to be in the F_0_ individuals that had missing genotypes at both markers. In region 3, the null allele genotypes were inferred using both the genotype calls and the intensity data. (A) Region 1 at 35.7 Mb on chromosome 1 observed in family 2. (B) Region 2 from 125.9−126.1 Mb on chromosome 1 observed in both families. (C) Region 3 at 166 Mb on chromosome 1 observed in both families. (D) Region 4 at 18.3 Mb on chromosome 3 observed in family 1.

**Figure 3:**
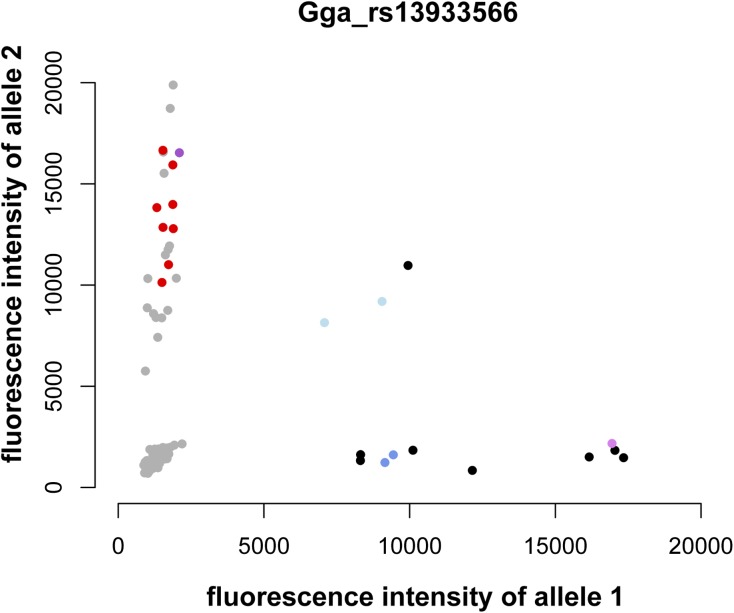
Intensity plot for a SNP in region 2. Intensity values are colored as follows: red, F_2_ individuals believed to have the null allele; black, other F_2_ individuals; dark purple, grandfather; light purple, grandmother; dark blue, parents thought to have the null allele; light blue, other parents; gray, additional individuals.

**Figure 4:**
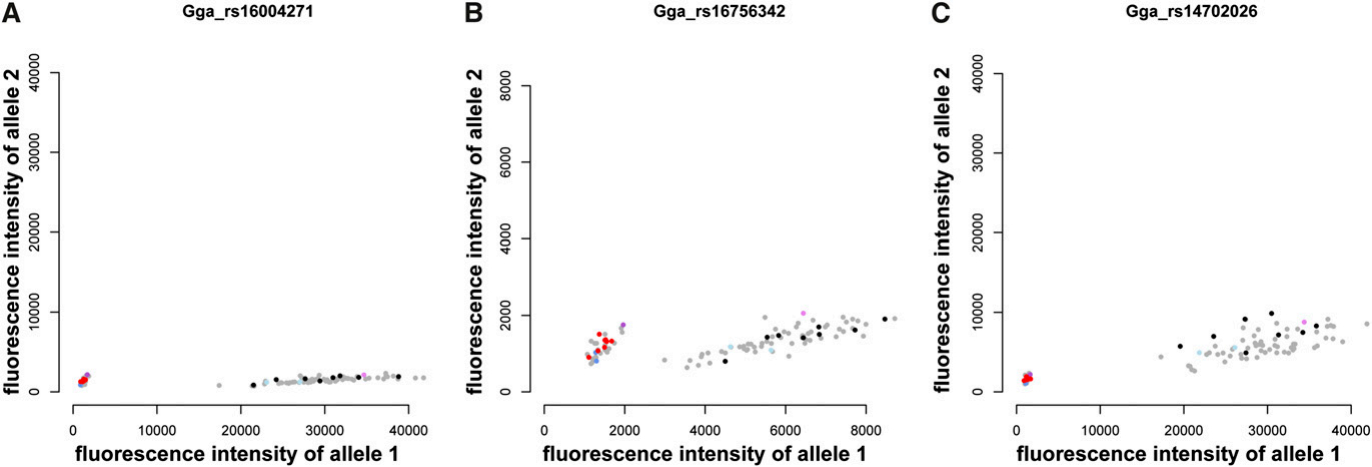
Intensity plot for three SNPs spanning region 3. Intensity values are colored as follows: red, F_2_ individuals that had missing genotypes at both SNPs in the 10K data; black, other F_2_ individuals; dark purple, grandfather; light purple, grandmother; dark blue, parents that had missing genotypes at both SNPs in the 10K data; light blue, other parents; gray, additional individuals (A) SNP preceding region 3. (B) SNP within region 3. (C) SNP succeeding region 3.

Cases of genotype incompatibilities that could not be explained by null alleles alone were treated as genotyping errors. These were split into instances where a parent and offspring were genotyped as different homozygotes and other errors. The remaining cases of possible null alleles were categorized into five classes (Figure 1) based on the number of genotype incompatibilities and how many genotyping errors would be needed to explain them. We define one genotyping incompatibility as an incompatibility between one parent and one of its offspring. The classes are (1) more than one incompatibility that cannot be explained by one genotyping error (Figure 1A), (2) more than one incompatibility that can be explained by one genotyping error (Figure 1B), (3) one incompatibility where no more incompatibilities are expected (*i.e.*, the incompatibility is between a parent and grandparent and the parents appear to have the same allele; Figure 1C), (4) one incompatibility where more incompatibilities would be expected (Figure 1D), and (5) more than five offspring with missing values (Figure 1E) or occurrence of both homozygotes and no heterozygotes in the offspring (Figure 1F). For class (5), there had to be more than one offspring with a nonmissing genotype, and when the classification was based on the absence of heterozygotes, there had to be more than one offspring with each homozygote genotype.

### Evidence from the two families

SNPs were given an overall classification from the results in the two families. When a SNP was in a different null allele class for the two families, if these were classes (1)−(4), the SNP was put in the numerically lower class. Because we subsequently found that class (4) SNPs were the least likely to have true null alleles, SNPs that were class (4) in one family and class (5) in the other were reported as class (5). SNPs with evidence for a null allele in one family and a genotyping error in the other family were placed in the null allele class. When a SNP had a genotyping error in both families and a parent and offspring were different homozygotes in one family but not the other, the SNP was recorded in the different homozygotes error category.

### Evaluating null allele classification

The different null allele classes identify SNPs with varied evidence for null alleles but they could have genotyping errors rather than null alleles. We found that very few SNPs classed as having genotyping errors showed errors in both families. The number of SNPs with detected errors was also considerably higher in family 1 than family 2. In contrast, in these data, null alleles should be fairly often seen in both families and be found at similar frequencies in the two families, because they share a grandfather and grandmother and the other grandmothers are from the same line. We therefore used a statistical approach to assess whether the null allele classes were likely to contain a high proportion of SNPs with genotyping errors. First, we compared the percentage of SNPs with possible null alleles in both families for each null allele class with the percentage of SNPs in the different homozygotes error class that showed genotyping errors in both families. Second, we compared the ratio of SNPs identified in family 1 to family 2 for each null allele class with the different homozygotes error class. A much greater percentage found in both families than for genotyping error and a ratio in family 1 to family 2 close to one is evidence that most of the SNPs in the null allele class do not have genotyping errors.

For null allele classes in which the ratio of SNPs in family 1 to family 2 was greater than one, we estimated the percentage of SNPs that had genotyping errors as follows. Treating the null allele class as a mixture of SNPs with null alleles, which occur at equal rates in the two families, and a fraction *x* of SNPs with different homozygotes genotyping errors, which occur at a ratio *r_e_* in family 1 to family 2, the ratio of SNPs from family 1 to family 2 in the null allele class is given by

$$r=\left(1-x\right)+xr_{e}.$$ (1)

Hence, *x* can be estimated by

$$x=\left(r-1\right)/\left(r_{e}-1\right).$$ (2)

### Putative deletions

We looked for possible null alleles at adjacent SNPs in the same family. A region was considered a putative deletion if a F_1_ parent and at least three F_2_ individuals showed evidence of linked null alleles at both SNPs. The size of the putative deletion was estimated as the distance between the SNPs.

### Denser genotyping and intensity data

On the basis of the segregation patterns at the SNPs showing putative deletions, we chose 22 F_2_ individuals for denser genotyping with the Illumina 60K chicken SNP chip described by Groenen *et al.* (2011). Eight F_2_ individuals were chosen from each family, such that half were expected to have each deletion. The shared grandfather, the shared grandmother, and the F_1_ parents of both families also were genotyped. The fluorescence intensity data for SNPs in and near the putative deletion regions were plotted along with results from 80 other individuals genotyped at the same time, to help identify genotype clusters. Plots should show three clusters for biallelic SNPs: individuals homozygous for one allele should fall close to one axis; individuals homozygous for the other allele, close to the other axis; and individuals heterozygous for the two alleles, on the *x = y* line in the center of the plot. We inspected the plots by eye for additional clusters. A second cluster near an axis but at lower values indicates individuals with one copy of an allele and one null allele and a cluster near the origin indicates individuals with two null alleles. If SNPs within and outside the original 10K interval showed null alleles, the extent of the candidate deletion was increased to the span of these SNPs.

### Genes in candidate deletion regions

The Ensembl (http://www.ensembl.org/) and UniProt (http://www.uniprot.org/) databases were accessed on 2/3/2012 to determine any genes within the candidate deletion regions and the possible functions of any protein products.

## Results

In total, we detected possible null alleles in 330 of the 10150 SNP markers (Table 1). Class (5) was largest and nearly all of these were cases in which many F_2_ individuals had missing values that could be due to two null alleles. It is unsurprising that this was the largest null allele class as missing genotypes can be observed regardless of the other genotypes in a family but whether there can be genotype incompatibilities will depend on which genotypes are present. Only two of the cases in class (5) showed an absence of heterozygote individuals and presence of both homozygotes in the offspring. Classes (2) and (4) were also large. Most of the SNPs in class (2) had a potential null allele in one F_1_ parent. Nearly all the cases in class (4) were due to one F_2_ offspring having a genotype that was incompatible with one of its parents. Class (1) contained 28 SNPs and for about half of these cases there appeared to be a null allele in both parents. In the others, the parent with the possible null allele and both its parents were homozygous for the same allele and therefore a single genotyping error cannot explain the observed genotype incompatibilities. Class (3) was the smallest class. These were SNPs with a single incompatibility between an F_1_ parent and one of its parents and both F_1_ parents appeared to have the same allele so a null allele could not cause incompatibilities between the parents and the F_2_ offspring.

**Table 1:** Number of SNPs with possible null alleles and genotyping errors

	Null Allele Class*^a^*					Genotyping Errors	
	(1)	(2)	(3)	(4)	(5)	Different Homozygotes	Other
Family 1	14	39	8	67	110	77	78
Family 2	16	28	7	7	134	5	16
Overall*^b^*	28	52	9	71	170	77	87
% in both families*^c^*	43	40	22	0	41	4	7
Ratio in family 1 to family 2	0.9	1.4	1.1	9.6	0.8	15.4	4.9
Estimated % error*^d^*	−	3	1	60	−		

SNP, single-nucleotide polymorphisms.

a The null allele classes are defined in the *Materials and Methods*.

b See *Materials and Methods* for a description of how the results were integrated across families.

c For the integrated results across families, the percentage of SNPs in the null allele classes that had evidence of null alleles in both families and the percentage of SNPs in the error categories that had genotyping errors in both families.

d Estimated percentage of SNPs in a null allele class that have genotyping errors rather than null alleles. Estimates were only possible when the ratio of SNPs for family 1 to family 2 was greater than one. See *Materials and Methods* for details.

There were 164 SNPs that we categorized as having genotyping errors and not null alleles (Table 1). Just less than half of these had a parent and offspring genotyped as different homozygotes. In null allele classes (1), (2), and (5), approximately 40% of the SNPs had evidence of null alleles in both families (Table 1). For class (3) this was 20%. None of the SNPs in class (4) were identified in both families. In the genotyping error categories, 4% and 7% of SNPs were recorded with errors in both families. More than 15 times more SNPs from family 1 than family 2 had apparent genotyping errors involving different homozygotes (Table 1). There were also nearly 10 times more SNPs from family 1 than family 2 in null allele class (4). The ratio of SNPs from family 1 to family 2 was slightly above one for null allele classes (2) and (3) and below one for classes (1) and (5). We estimated that 60% of the SNPs in class (4) had genotyping errors rather than null alleles (Table 1). The estimates for class (2) and (3) were only 3% and 1%, respectively.

Four putative deletions were found from the 10K genotypes, which were on chromosomes 1 and 3 (Table 2). Two of these were identified by class (5) null alleles, one by class (2) null alleles, and one by a class (1) and a class (2) null allele. The estimated sizes of the putative deletions ranged from 5 to 157 kb. Two of the regions were detected in both families, one only in family 1, and one only in family 2. The genotypes for the regions are shown in Figure 2, with the suspected null alleles indicated. For region 2 the putative deletion is from the Red Junglefowl and for region 4, from the White Leghorn. For regions 1 and 3 the origin of the potential deletion is not conclusive from the genotypes. For region 1, the grandmothers had missing values suggesting that the deletion is from the White Leghorn. For region 3, the subsequent intensity data indicated that the deletion was homozygous in the Red Junglefowl individual but also occurred in the White Leghorn.

**Table 2:** Putative deletion regions identified from 10K SNP genotypes

Region	Chrom	SNPs	Null Allele Classes	First Position, Mb	Second Position, Mb	Estimated Size, kb	Family
1	1	rs14809025, rs13848816	5	35.74	35.74	5.3	2
2	1	rs14882681, rs13933665	2	125.89	126.05	157.4	1, 2
3	1	rs16689692, rbl2025	5	165.95	165.98	33.6	1, 2
4	3	rs14320118, rs15280151	1, 2	18.31	18.33	15.4	1

SNP, single-nucleotide polymorphisms.

There was support for two of the putative deletions from the 60K SNP chip data. They were the regions that were detected in both families. For region 2, there were five new SNPs with positions inside the 10K interval. In the intensity plot for the second of these SNPs, the grandfather and F_2_ individuals inferred to have the deletion clustered along the *y*-axis and the F_1_ parents thought to have the deletion were close to the *x*-axis (Figure 3). Therefore, in this subset of individuals, only one allele was detected and it was a different allele in the grandfather and F_2_ individuals inferred to have the deletion than in the parents. These data show a null allele consistent with the results from the 10K SNPs and support a deletion. However, there were some conflicting findings. The intensity data from the first and third SNPs showed that the individuals inferred to have the deletion were heterozygous and therefore did not have a null allele at these SNPs. Because of these conflicting findings, we did not change our estimate of the extent of this candidate deletion.

The 60K SNP chip contained one new marker located between the 10K markers in region 3. The intensity plot for this SNP, and the closest SNP on either side had a distinct cluster of individuals near the origin and the remaining individuals grouped together at greater x-values (Figure 4). The cluster close to the origin contained the grandfather (who had missing genotypes at both SNPs in the 10K data), the F_2_ individuals with missing genotypes at both SNPs in the 10K data and the parents from each family that had missing genotypes at both SNPs in the 10K data. Hence, the intensity data show that these individuals have two null alleles. It follows, and is supported by their similar intensity values, that the grandmother, other parents and remaining F_2_ individuals all have one null allele. The intensity data for these three additional markers support a deletion in this region and show that the deletion is in both the Red Junglefowl and the White Leghorn, with the Red Junglefowl being homozygous for the deletion and one White Leghorn female being heterozygous for the deletion. Based on the 10K data, at least one other White Leghorn female is also heterozygous for the deletion. We increased the estimated extent of this candidate deletion to 165.93−165.98 Mb with an estimated size of 55.6 kb.

There were no new markers within region 1 on the 60K SNP chip. The intensity plots of the closest marker on either side of region 1 gave no indication of null alleles. Additional data would therefore have to be collected to evaluate this region. For region 4, there was one new marker on the 60K SNP chip located between the 10K markers. The intensity plots for this marker and the nearest marker on either side showed that the F_2_ individuals thought to carry the deletion were heterozygous and do not support a deletion in this region.

Region 2 covers the first seven out of nine exons of gene *GLRA2* (Ensembl). However, the span of the potential deletion is uncertain because of the conflicting results from the 60K SNP data. The gene has a single transcript *ENSGALT00000026747*. The protein is uncharacterized but clusters with 90% identity to glycine receptor subunit alpha-2 proteins in human, mouse and rat, which are involved in inhibitory neurotransmission (UniProt). Region 3 covers the complete length of gene *ENSGALG00000014545*, which has a single transcript, *ENSGALT00000023466* (Ensembl). The protein is uncharacterized but clusters with 90% identity to zinc finger RNA-binding proteins in human, orangutan, mouse and rat (UniProt).

## Discussion

We detected a large number of possible null alleles by examining ~10K SNP genotypes for genotype incompatibilities and missing values in two large chicken families. The suspected null alleles were grouped into different classes based on the type of evidence for null alleles. We used the findings across families to assess whether the classes detected true null alleles. From the null allele results, we identified four potential deletions segregating in the population. Two of these were supported by new genotyping results from a 60K SNP chip, one was not supported by the new data and in one case the denser chip provided no additional information. The two candidate deletions were on chromosome 1. Both were present in the Red Junglefowl line and one was also in the White Leghorn Line. Each candidate spans a protein-coding region.

We evaluated the null allele classes by measuring the percentage of SNPs that were identified with possible null alleles in both families and the ratio of SNPs found from family 1 to family 2. Both approaches indicate that a high proportion of SNPs in classes (1)-(3) and class (5) have true null alleles but suggest that many class (4) SNPs have genotyping errors. Class (4) consists of cases with one genotype incompatibility, predominately between an F_1_ parent and one of the F_2_ offspring, where more incompatibilities would be expected if the parent had a null allele. Class (3) also consists of SNPs with one incompatibility but the incompatibility is between an F_1_ parent and F_0_ grandparent and because of the genotypes in the family a null allele could not cause incompatibilities in the F_2_ offspring. SNPs in both these classes could have a single genotyping error rather than a null allele. However, the distribution of SNPs between families in these two classes was very different. For class (3), 22% of SNPs had evidence of null alleles in both families compared with 0 in class (4). The ratio of SNPs found in family 1 to family 2 was just greater than 1 for class (3) and nearly 10 for class (4). By comparing this ratio to the value for different homozygote errors, we estimated that only 1% of the SNPs in class (3) had genotyping errors. We were able to distinguish classes (1)−(4) because we had genotypes for multiple offspring. In studies with a single offspring, such as those in humans, null alleles can only generate one genotype incompatibility. Our results suggest that null allele detection is more accurate when multiple offspring are genotyped and cases with a genotype incompatibility between a parent and only one of the offspring are excluded.

Null allele class (5) predominantly contained SNPs with missing genotypes and we had expected this class to have a high false-positive rate because there was no direct evidence for a null allele. Instead, our statistical assessment indicated that most class (5) SNPs had true null alleles. It was also class (5) SNPs that identified one of the candidate deletions. Class (5) probably had a high rate of true null alleles because we had data for multiple offspring and only included SNPs based on missing genotypes when at least five individuals had missing genotypes. Random genotyping error is unlikely to cause several missing genotypes. However, there could be multiple missing genotypes if there were technical problems with the genotyping assay. This type of genotyping failure would not be detected by the measures we used to assess the null allele classes if it produced missing values in both families. To try to reduce the possibility that class (5) SNPs were due to problems with the genotyping assay, we excluded instances where all, or all but one, F_2_ individuals had missing genotypes. The average number of individuals that had genotype calls for SNPs in class (5) was seventeen per family, which suggests that assay problems are not a general explanation for these cases.

The estimated percentage of SNPs with null alleles after excluding class (4) was 2.6%. Adding class (4) to the error categories, the estimated percentage of SNPs with genotyping errors was 2.3%. The actual values of both may be higher because not all genotyping errors and probably not all null alleles would have been detectable given the genotypes in a family. Some null alleles in the F_0_ individuals will not have been transmitted to the F_1_ parents. Null alleles in the grandfather had a higher chance of being transmitted than null alleles in one of the grandmothers because he fathered all the F_1_ parents. Although we have estimated the error rate within the null allele classes to be low, some of the possible null alleles will be genotyping errors. Including more related families should increase the number of null alleles found because it increases the chance of the alleles being transmitted and the alleles may occur with a different set of genotypes. However there will be diminishing returns from adding families as the number of families is increased. The number of SNPs with genotyping errors will increase if more families are added and this increase should be independent of the number of families already included. Our results suggest that a relatively high proportion of genotype inconsistencies in chicken SNP data could be due to null alleles and therefore there may be useful information in these data. We estimated that nearly 40% of SNPs in which a parent and offspring were genotyped as different homozygotes had null alleles. When only a single marker shows evidence of a null allele, it is not possible to know whether this is due to a deletion or an additional SNP in the primer-annealing site that interferes with the amplification. In this article, we only consider null alleles as a sign of a potential deletion when two adjacent SNPs show evidence of null alleles in the same individuals.

To gain support for the putative deletions we identified from our null allele results, we genotyped selected individuals with a 60K SNP chip. The intensity data from the 60K chip provided support for two of the potential deletions. However, for one of these, region 2, there were conflicting results from the SNPs located within the 10K interval. One SNP showed null alleles in the individuals thought to have the deletion, but for two of the other SNPs these individuals were heterozygous and therefore did not have null alleles. Overall, we believe that there is sufficient evidence to consider this region as a candidate deletion. Possible explanations for the discrepancies in results between SNPs are that the relative positions of the 60K and 10K SNPs are incorrect in the consensus sequence, that the probes for those SNPs not showing null alleles also target another position in the genome, or that there is a more complex rearrangement in this region. Quantitative PCR with a series of primers flanking and within region 2 would confirm if there is a deletion and help in determining its size and understanding the different findings across SNPs.

The data used in this study are from two families of an F_2_ intercross between a domesticated White Leghorn chicken line and its wild ancestor, the Red Junglefowl. The founder lines differ considerably in a number of traits including growth, egg production and behavior (Kerje *et al.* 2003; Schutz *et al.* 2004). One of our candidate deletions were detected only in the Red Junglefowl and the other was homozygous in the Red Junglefowl but segregating in the White Leghorn founders, and therefore they may contribute to the observed phenotypic differences between these lines. A genome-wide scan for QTL affecting growth in this intercross detected 22 loci (Carlborg *et al.* 2003; Kerje *et al.* 2003). Our candidate deletions are each located within 9 cM of one of these QTL, based on the consensus linkage map (Groenen *et al.* 2009). Region 2 was near an epistatic QTL that interacted with a locus on chromosome 14 to affect hatch weight. Region 3 was located near a QTL that affected post-hatch body weight at four different ages and growth between these ages. To investigate these candidates further, the deletions need to be confirmed and their breakpoints determined. After validation, the next step would be to genotype the whole intercross population for the deletion and directly test for an association with body weight.

We suggest that our method for detecting potential deletions could be a useful complement to linkage analysis of intercross data. Linkage analysis generally identifies fairly broad QTL regions that contain numerous genes. Deletions that are close to QTL are possible candidates for the variants underlying QTL effects. Usually in CNV studies, many variants are detected but it is difficult to evaluate which could be functionally important. When linkage regions have been associated with a trait, there is incidental evidence that putative deletions in these regions are functional. Intercross data have some advantages for identifying null alleles and inferring deletions from them. The sets of related families can increase confidence in possible null alleles that are detected in several families. For both our candidate deletions that were supported by the 60K data, null alleles were implicated in both families. There is also data available from grandparents. Checking that grandparental genotypes are consistent with null alleles is an additional filter to improve accuracy.

High-density SNP arrays have been used for CNV discovery in humans for several years. These studies are usually carried out in populations of unrelated individuals and require consistent signals at multiple SNPs to call a CNV. Lower density SNP arrays have recently become available for a number of domesticated plants and animals. The density in these arrays is probably too low to detect moderate-sized deletions using the population-level approaches applied to human data. Here, we have shown that with large families, where the pattern of null alleles in the pedigree can be used, data from these lower density arrays can identify null alleles at adjacent markers that could be deletions. We considered linked null alleles at two adjacent SNPs sufficient to infer a candidate deletion and two of our four potential deletions were supported by additional genotyping data. Some of the single SNPs with possible null alleles, particularly those in class (1) and (2) that were identified in both families, may also be due to deletions and it would be interesting to investigate these regions with denser SNP data. Despite only requiring two SNPs to show evidence of null alleles, both our candidate deletions were estimated to be larger than 50 kb. More, and shorter, deletions will be detectable as higher density arrays are produced, reflecting the increase in reported CNV numbers with array density in human genetics.

## Supplementary Material

Supporting Information
