# Supplementary material for: Identification of Null Alleles and Deletions from SNP Genotypes for an Intercross Between Domestic and Wild Chickens
Source: G3 (Bethesda). 2013 Aug 1;3(8):1253–60. doi: 10.1534/g3.113.006643 (PMC3737165; doi:10.1534/g3.113.006643)
Supplement: Supporting Information [file supp_3_8_1253__index.html]

Identification of Null Alleles and Deletions from SNP Genotypes for an Intercross Between Domestic and Wild Chickens — Supporting Information 

# Identification of Null Alleles and Deletions from SNP Genotypes for an Intercross Between Domestic and Wild Chickens

## Supporting Information for Crooks *et al.*, 2013

**Files in this Data Supplement:**

- Supporting Information - File S1 (PDF, 259 KB)
- File S1 - Data files (.zip, 248 KB)
