# Supplementary material for: Identification of Null Alleles and Deletions from SNP Genotypes for an Intercross Between Domestic and Wild Chickens
Source: G3 (Bethesda). 2013 Aug 1;3(8):1253–60. doi: 10.1534/g3.113.006643 (PMC3737165; doi:10.1534/g3.113.006643)
Supplement: Supporting Information [file supp_g3.113.006643_006643SI.pdf]

G3 · Genes | Genomes | Genetics

Identification of null alleles and deletions from SNP genotypes for an intercross between domestic and wild chickens

Lucy Crooks[*1], Örjan Carlborg[§], Stefan Marklund[§], Anna M Johansson[†]

[*] Department of Cell and Molecular Biology, Uppsala University, Uppsala, SE-75124, Sweden
[§] Department of Clinical Sciences, Swedish University of Agricultural Sciences, Uppsala, SE-756 51, Sweden
[†] Department of Animal Breeding and Genetics, Swedish University of Agricultural Sciences, Uppsala, SE-75007, Sweden
[1] Present address: Wellcome Trust Sanger Institute, Hinxton, Cambridge, CB10 1HH, United Kingdom

DOI: 10.1534/g3.113.006643

File S1

Data Files

File S1 is available for download at http://www.g3journal.org/lookup/suppl/doi:10.1534/g3.113.006643/-/DC1.

pedigree.txt
File with one line per sample
Individuals grouped in families
First row for each family gives number of F_2 individuals in family
Second to fifth rows give data on grandparents
Sixth and seventh rows, data on parents
Remaining rows, data on F_2 individuals
Columns are id, sire id, dam id, sex and line (for founders)
Sex is recorded as 1 for males and 2 for females
Line is recorded as 1 for Red Junglefowl and 2 for White Leghorn

genotypes.txt
Data from the 10K SNPs
Header with SNP names
Ids in first column, genotypes in subsequent columns
One line per sample
Genotypes coded as 11, 12, 22; missing genotypes are represented by 00

intensity_data_region_x.txt
Data from the 60K SNPs in regions identified by 10K SNPs
Columns are SNP name, sample id, X raw intensity and Y raw intensity
For region 2, five SNPs in the interval determined from 10K SNPs are given
For regions 3 and 4, the SNP in the interval determined from 10K SNPs and the two flanking SNPs are given
There were no SNPs in the interval determined from 10K SNPs for region 1

###first_marker_per_chromosome.txt
File with the first marker on each chromosome, i.e. marker number 1 is the first marker on chromosome 1, marker number 1927 is the first marker on chromosome 2, marker number 3032 is the first marker on chromosome 3 and so on.
